# *In Vitro* Oxygen-Glucose Deprivation-Induced Stroke Models with Human Neuroblastoma Cell- and Induced Pluripotent Stem Cell-Derived Neurons

**DOI:** 10.1155/2020/8841026

**Published:** 2020-10-29

**Authors:** Miia Juntunen, Sanna Hagman, Anaick Moisan, Susanna Narkilahti, Susanna Miettinen

**Affiliations:** ^1^Adult Stem Cell Group, Faculty of Medicine and Health Technology, Tampere University, Tampere, Finland; ^2^Research, Development and Innovation Centre, Tampere University Hospital, Tampere, Finland; ^3^Neuro Group, Faculty of Medicine and Health Technology, Tampere University, Tampere, Finland; ^4^Cell Therapy and Engineering Unit, EFS Auvergne Rhône Alpes, 38330 Saint Ismier, France

## Abstract

Stroke is a devastating neurological disorder and one of the leading causes of mortality and disability. To understand the cellular and molecular mechanisms of stroke and to develop novel therapeutic approaches, two different *in vitro* human cell-based stroke models were established using oxygen-glucose deprivation (OGD) conditions. In addition, the effect of adipose stem cells (ASCs) on OGD-induced injury was studied. In the present study, SH-SY5Y human neuroblastoma cells and human induced pluripotent stem cells (hiPSCs) were differentiated into neurons, cultured under OGD conditions (1% O_2_) for 24 h, and subjected to a reperfusion period for 24 or 72 h. After OGD, ASCs were cocultured with neurons on inserts for 24 or 72 h to study the neuroprotective potential of ASCs. The effect of OGD and ASC coculture on the viability, apoptosis, and proliferation of and axonal damage to neuronal cells was studied. The results showed that OGD conditions induced cytotoxicity and apoptosis of SH-SY5Y- and hiPSC-derived neurons, although more severe damage was detected in SH-SY5Y-derived neurons than in hiPSC-derived neurons. Coculture with ASCs was protective for neurons, as the number of dead ASC-cocultured neurons was lower than that of control cells, and coculture increased the proliferation of both cell types. To conclude, we developed *in vitro* human cell-based stroke models in SH-SY5Y- and hiPSC-derived neurons. This was the first time hiPSCs were used to model stroke *in vitro*. Since OGD had different effects on the studied cell types, this study highlights the importance of using several cell types in *in vitro* studies to confirm the outcomes of the study. Here, ASCs exerted a neuroprotective effect by increasing the proliferation and decreasing the death of SH-SY5Y- and hiPSC-derived neurons after OGD.

## 1. Introduction

Stroke is a devastating disease that is a leading cause of long-term disability and death [1]. It is caused by compromised blood supply to the brain, leading to oxygen and glucose deficiencies in the central nervous system (CNS). A lack of energy causes excitotoxicity, mitochondrial dysfunction, free radical release, protein misfolding, and inflammatory responses, eventually leading to neural injury. Consequently, neuroinflammatory responses that lead to activation of immune cells and upregulation of cytokines, chemokines, and reactive oxygen species are triggered [2]. Currently, there are two approved treatments for ischemic stroke: thrombolytic therapy using tissue plasminogen activator [3, 4] and mechanical thrombectomy [4]. Their use is limited to a short therapeutic time window [4], and therefore, the majority of stroke patients are not able to receive such treatments. Therapies targeting later time windows are urgently needed [5].

Animal models of stroke have played a substantial role in elucidating the pathogenetic mechanisms of stroke. However, novel therapeutic approaches for stroke have repeatedly failed in clinical phase studies after having success in preclinical animal models [5, 6]. The high failure rate in clinical trials may be due to the numerous macrostructural, cellular, and molecular discrepancies that exist between rodent and human brains [7]. *In vitro* human cell-derived models are considered to be advantageous for overcoming these challenges, revealing the pathological mechanisms of stroke and therefore developing new therapeutic drugs. Currently, the oxygen-glucose deprivation- (OGD) induced model is the most relevant and commonly used *in vitro* model mimicking stroke [7]; however, studies have shown wide variability in the degree of injury [8]. The majority of OGD studies have used either primary rodent neuronal cells or neuroblastoma cell lines, such as the SH-SY5Y cell lines [9]. The SH-SY5Y cell line is a human-derived cell line that has been used for research on various neurological disorders, such as Parkinson's disease, Alzheimer's disease, ischemia, and amyotrophic lateral sclerosis [8, 10–12]. Since SH-SY5Y cells are of cancerous origin, they have several genetic aberrations; therefore, their use in *in vitro* models has been criticized [7]. Thus, *in vitro* nonneoplastic human cell-based models need to be developed. Human induced pluripotent stem cell- (hiPSC) derived neural cells show great promise for studying neurological diseases because they are expendable cellular sources of neuronal cells, which are naturally hard to access [13, 14]. To our knowledge, there have been no reports on using hiPSC-derived neurons to model stroke *in vitro*.

Transplantation of mesenchymal stem cells (MSCs) represents a new potential therapeutic strategy for stroke [15]. In previous *in vivo* animal studies, transplantation of MSCs has been shown to promote functional recovery and reduce lesion size [16, 17], and these cells have already been utilized in clinical phase studies with varying results [18]. The mechanisms of action of MSCs are not known, but their restorative functions are suggested to be mediated by a paracrine effect. MSCs secrete various neurotrophic, angiogenic, and immunoregulatory factors, thereby suppressing inflammation and promoting angiogenesis, neurogenesis, remyelination, and axonal plasticity [19]. It is also noteworthy that endogenous neural stem cells can secrete multiple factors that are able to beneficially regulate neurogenesis and modulate inflammatory responses after CNS damage [20].

Adult MSCs can be harvested from the bone marrow (BM-MSCs) and adipose tissue, among other tissues of mesenchymal origin [21]. Adipose tissue-derived stem cells (ASCs) present multiple advantages due to their higher yield from donors and because they require less invasive harvesting methods than BM-MSCs [21]. In addition, MSCs can be expanded with human platelet lysate instead of fetal bovine serum to avoid immunological reaction to xenogenic substances [22, 23]. ASCs have been shown to have a beneficial effect on stroke recovery in animal models [24–26]. We, among others, have shown that ASCs improve behavioral recovery [24, 26] and reduce the death of neural cells in *in vivo* models [24]. However, ASC therapy has different results, especially in animal models with induced comorbidities such as hypertension [27, 28] and diabetes [27], in which beneficial effects of ASCs are not observed.

In this study, we developed two *in vitro* human cell-based models of ischemic stroke using neurons differentiated from the neuroblastoma cell line SH-SY5Y and hiPSCs and optimized an OGD protocol for use in cell models. In addition, we studied the paracrine neuroprotective effect of human ASCs against OGD-induced injury in these two *in vitro* models.

## 2. Materials and Methods

### 2.1. Differentiation of SH-SY5Y Neuroblastoma Cells into Neurons

The human neuroblastoma cells SH-SY5Y (ATCC) were thawed and cultured in basic medium including Eagle's minimum essential medium (EMEM, Sigma-Aldrich) supplemented with 15% heat-inactivated fetal bovine serum (FBS, Thermo Fisher Scientific), 1% penicillin-streptomycin (P/S, Lonza), and 1% HyClone L-glutamine (GE Healthcare Bio-Sciences Austria GmbH). SH-SY5Y cells were differentiated into neuronal cells by using the protocol described by Shipley et al. [9] with moderate modifications. Briefly, SH-SY5Y cells were detached from cell culture flasks with TrypLE Select (Thermo Fisher Scientific), suspended in basic medium and plated into 6-well plates at a density of 10,000 cells/cm^2^ (Nunc). The next day, the medium was changed to neuronal differentiation (ND) 1 medium supplemented with 2.5% FBS and 10 *μ*M retinoic acid (RA, Sigma), and the cells were cultured for 7 days. Thereafter, the cells were cultured in ND2 medium supplemented with 1% FBS and 10 *μ*M RA for 3 days. After that, the SH-SY5Y cells were detached with TrypLE Select and plated in 15 *μ*g/ml laminin (LN521, Biolamina)-coated 24-well plates (Nunc) at a density of 25,000 cells/cm^2^. The next day, the medium was changed to ND3, which was composed of neurobasal medium (Thermo Fisher Scientific) supplemented with 2% B-27 (Thermo Fisher Scientific), 20 mM potassium chloride (Merck), 1% P/S, 2 mM GlutaMAX (Thermo Fisher Scientific), 50 ng/ml brain-derived neurotrophic factor (BDNF, R&D Systems), 2 mM dibutyryl cyclic AMP (db-cAMP, Sigma), and 10 *μ*M RA. SH-SY5Y cells were cultured under these conditions for 11 days; thus, altogether, differentiation was performed for 20 days prior to the start of OGD treatment. Hereafter, differentiated SH-SY5Y cells are referred to as SH-SY5Y-neurons.

### 2.2. Human Pluripotent Stem Cells and Differentiation of Neurons

The hiPSC line 10212.EURCCs [29] was generated with Sendai virus technology (Life Technologies) [30] at the Faculty of Medicine and Health Technology (MET), Tampere University, Finland. MET has received supportive statements from the regional ethics committee of Pirkanmaa Hospital District for the derivation, culture, and differentiation of hiPSCs (R08070). Informed consent was obtained from patients who provided cell samples. hiPSCs were expanded in a feeder-free culture system as described previously [31]. Cortical neurons were differentiated as previously described [32]. Briefly, the basal medium consisted of 1 : 1 DMEM/F12 with GlutaMAX, neurobasal medium, 0.5% N_2_, 1% B-27 with RA, 0.5 mM GlutaMAX, 0.5% NEEA, 50 *μ*M 2-mercaptoethanol (all purchased from Thermo Fisher Scientific), 2.5 *μ*g/ml insulin (Sigma), and 0.1% P/S (Thermo Fisher Scientific). For neural induction, the basal medium was supplemented with 100 nM LDN193189 (Sigma) and 10 *μ*M SB431542 (Sigma) for 12 days. Thereafter, the cells were cultured in basal medium supplemented with 20 ng/ml fibroblast growth factor-2 (FGF2, R&D Systems) for 13 days. Then, the cells were cultured in maturation medium, which consisted of basal medium supplemented with 20 ng/ml BDNF, 10 ng/ml glial-derived neurotrophic factor (R&D Systems), 500 *μ*M db-cAMP, and 200 *μ*M ascorbic acid (Sigma) for 7 days to promote the maturation of neurons. On day 32, the cells were plated in 24-well plates (Nunc) at a density of 100,000 cells/cm^2^ and cultured for 7 days prior to OGD treatment. Differentiated hiPSCs are hereafter referred to as hiPSC-neurons.

### 2.3. Adipose Stem Cells

To study the effect of ASCs on neuronal cells, human ASCs (Master Cell Bank/Stock no. 1—Donor RESSTORE01, Batch no. 591133643763) were cultured in Alpha MEM (Gibco) supplemented with 5% human platelet lysate (Stemulate, Cook Medical) and 1% P/S. ASCs were isolated and cultured as previously described [26, 27]. The ASC phenotype was analyzed by flow cytometry (FACSAria Fusion Cell Sorter, BD Biosciences) and was found to reflect a typical MSC immunophenotype featuring expression (>95%) of the surface markers CD73, CD90, and CD105 and no expression (<2%) of CD11a, CD19, CD34, CD45, and HLA-DR [26].

### 2.4. Oxygen-Glucose Deprivation and Reperfusion

**Figure 1:**
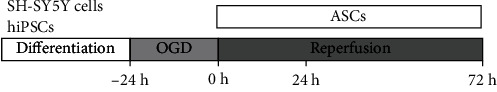
Timeline of the study. SH-SY5Y neuroblastoma cells and human induced pluripotent stem cells (hiPSCs) were differentiated into neuronal cells. The cells were subjected to oxygen-glucose deprivation (OGD) in 1% O_2_ without glucose for 24 h. Then, the cells were transferred back to normoxic (19% O_2_) conditions with medium containing glucose for 24 or 72 h (reperfusion). To study the effect of human adipose stem cells (ASCs), ASCs and neurons were cocultured after OGD for 24 or 72 h. ASCs were cultured on inserts on the top of neurons.

To model ischemic stroke *in vitro*, cells were first washed with glucose-free medium. Thereafter, SH-SY5Y-neurons were incubated in glucose-free ND3 medium, and hiPSC-neurons were incubated in glucose-free DMEM in a humidified oxygen control CO_2_ incubator (HeraCell, Thermo Fisher Scientific) with 1% O_2_, 5% CO_2_, and 94% N_2_ for 24 h at 37°C. Immediately after OGD, cells were reperfused by removing the medium and replacing it with ND3 for SH-SY5Y-neurons and maturation medium containing glucose for hiPSC-neurons, and the cells were incubated for 24 or 72 h at 37°C in 95% air/5% CO_2_ (Figure 1). Control SH-SY5Y-neurons and hiPSC-neurons were washed and incubated in ND3 medium and maturation medium containing glucose, respectively, under normoxic conditions for 24 h in 95% air/5% CO_2_ (HeraCell).

### 2.5. Coculture of Neurons and ASCs

The effects of ASCs on OGD-treated SH-SY5Y- and hiPSC-neurons during reperfusion were assessed in cocultures. ASCs (10,000 cells/insert, density of 30,000 cells/cm^2^) were plated on ThinCert™-TC inserts (pore size 0.4 *μ*m, Greiner Bio-One) and incubated with SH-SY5Y- or hiPSC-neurons for 24 or 72 h at 37°C and 95% air/5% CO_2_ (HeraCell). The experimental design is presented in Figure 1.

### 2.6. Immunocytochemical Staining

Immunocytochemical staining was performed as previously described [33]. The primary antibodies included dendritic marker microtubule-associated protein 2 (MAP2, chicken, 1 : 4000, NB300-213, Novus), microtubulin marker *β*-tubulin_III_ (mouse, 1 : 1000, T8660, Sigma), apoptosis marker cleaved caspase-3 (cl-Casp3, rabbit, 1 : 400, 9664, Cell Signaling), and proliferation marker Ki-67 (rabbit, 1 : 800, AB9260, Millipore). The secondary antibodies included Alexa Fluor 488-conjugated donkey anti-rabbit (1 : 400), Alexa Fluor 568-conjugated donkey anti-mouse (1 : 400), and Alexa Fluor 647-conjugated goat anti-chicken (1 : 200, all from Thermo Fisher Scientific). Cell samples were mounted with ProLong™ Gold Antifade Mountant with DAPI (Thermo Fisher Scientific). Images were acquired with an Olympus IX51 microscope equipped with an Olympus DP30BW camera (Olympus Corporation). CellProfiler [34] and CellProfiler Analyst [35] were used for image analysis.

### 2.7. CyQuant Analysis

The number of cells was analyzed based on the amount of DNA in the samples using a CyQuant™ cell proliferation assay kit (Molecular Probes, Invitrogen™) as previously described [36]. Briefly, the medium was discarded, and the cells were rinsed with DPBS and 0.1% Triton-X-100 (Sigma-Aldrich) and frozen at -80°C until analysis. Fluorescence was measured with a microplate reader (Victor 1429 Multilabel Counter, Wallac) at 480/520 nm.

### 2.8. qRT-PCR

Quantitative real-time PCR (qRT-PCR) analysis of *tubulin β-III* (*TUBB3*) and *growth-associated protein 43* (*GAP43*) and for the endogenous control *human acidic ribosomal phosphoprotein P0* (*RPLP0*) was performed as previously described [37]. Briefly, total RNA was isolated by using the NucleoSpin RNA® II kit (Macherey-Nagel) according to the manufacturer's instructions, and RNA samples were reverse transcribed into cDNA using the High-Capacity cDNA Reverse Transcriptase Kit (Applied Biosystems). Gene expression was analyzed with Power SYBR Green PCR Master Mix (Applied Biosystems) on an ABI Prism 7300 real-time PCR system (Thermo Fisher Scientific). The primers used for expression analysis were as follows: *TUBB3* forward 5′-GCCTTCCTGCACTGGTACAC-3′, reverse 5′-TACATCTCGCCCTCTTCCTC-3′; *GAP43* forward 5′-AGAGCAGCCAAGCTGAAGAG-3′, reverse 5′-TCTTGGTCAGCCTCAGGTTC-3′; and *RPLP0* forward 5′-AATCTCCAGGGGCACCATT-3′, reverse 5′-CGCTGGCTCCCACTTTGT-3′ (Oligomer Oy). The expression levels of *TUBB3* and *GAP43* were normalized to the expression level of *RPLP0*, and the relative expression of the studied genes was calculated using a mathematical model as previously described [38].

### 2.9. Viability Staining

A LIVE/DEAD Cell Viability/Cytotoxicity kit for mammalian cells (Thermo Fisher Scientific) was used to evaluate the viability of neuronal cells. The cells were incubated for 30 min at 37°C with 0.1 *μ*M green fluorescent calcein-AM to detect live cells and with 0.5 *μ*M red fluorescent ethidium homodimer-1 (EthD-1) to detect dead cells. The samples were imaged immediately with an Olympus IX51 microscope equipped with an Olympus DP30BW camera. For all staining experiments, four images per well were taken and used for image analysis. CellProfiler [34] software was used to perform image analysis as previously described [39], where the areas of calcein-AM- and EthD-1-positive cells in the images were determined from.

### 2.10. Statistics

An independent *t*-test was used for normally distributed data. A *p* value less than 0.05 was considered significant. If Bonferroni correction was applied, a *p* value less than 0.025 was considered significant. All data are presented as the mean ± standard error of the mean (SEM). Statistical analysis was performed using IBM SPSS Statistics 25 (IBM), and graphs were generated using GraphPad Prism 5.02 (GraphPad Software, Inc.).

## 3. Results

### 3.1. ASC Treatment Reduced OGD-Induced Cytotoxicity in SH-SY5Y- and hiPSC-Neurons

**Figure 2:**
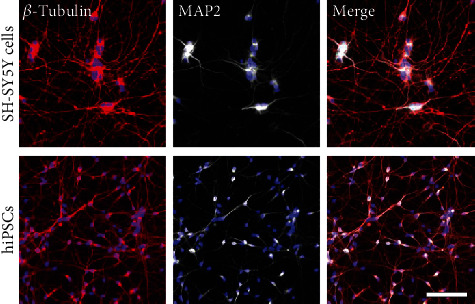
SH-SY5Y- and hiPSC-neurons after neuronal differentiation. Representative staining for *β*-tubulin_III_ and MAP2 confirming the presence of neuronal populations in the cultures. Scale bar, 100 *μ*m.

To model stroke *in vitro*, SH-SY5Y- and hiPSC-neurons were exposed to OGD for 24 h followed by reperfusion for 24 h or 72 h. Additionally, the paracrine effect of ASCs on injured neurons and control cells was studied. First, differentiated SH-SY5Y-neurons and hiPSC-neurons were characterized by staining for the neuronal marker *β*-tubulin_III_ and the dendritic marker MAP2, which were both expressed at high levels after neuronal differentiation in both cell types. After differentiation, SH-SY5Y cells and hiPSCs adopted a more neuronal cell-like morphology with long and branched neurites (Figure 2).

**Figure 3:**
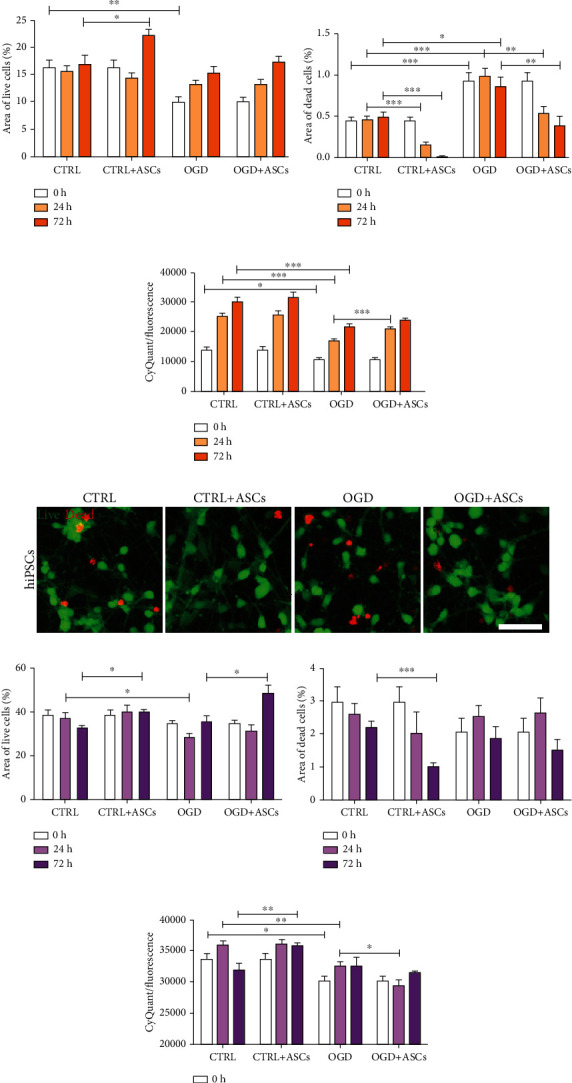
Cell viability and cytotoxicity of SH-SY5Y- and hiPSC-neurons after OGD and ASC coculture. (a, e) Representative images of live/dead staining after 72 h of reperfusion in SH-SY5Y-neurons (a) and hiPSC-neurons (e). Green = live cells/calcein-AM; red = dead cells/EthD-1. Scale bar, 100 *μ*m. (b) Area of live SH-SY5Y-neurons and (f) hiPSC-neurons. (c–g) Area of dead SH-SY5Y-neurons (c) and (g) hiPSC-neurons. For quantification of live and dead cells, images were analyzed from two to three wells/group, and four images were taken from each well (one experiment for SH-SY5Y-neurons and representative data of two separate experiments for hiPSC-neurons). (d) Analysis (CyQuant) of the number of SH-SY5Y-neurons (*n* = 3/group, data from four independent experiments) and (h) hiPSC-neurons (*n* = 3/group, representative data of two separate experiments). All statistical analyses were performed using an independent *t*-test, statistical significance: ^∗∗∗^*p* < 0.001, ^∗∗^*p* < 0.01, and ^∗^*p* < 0.05. All quantitative data are presented as the mean ± SEM.

**Figure 4:**
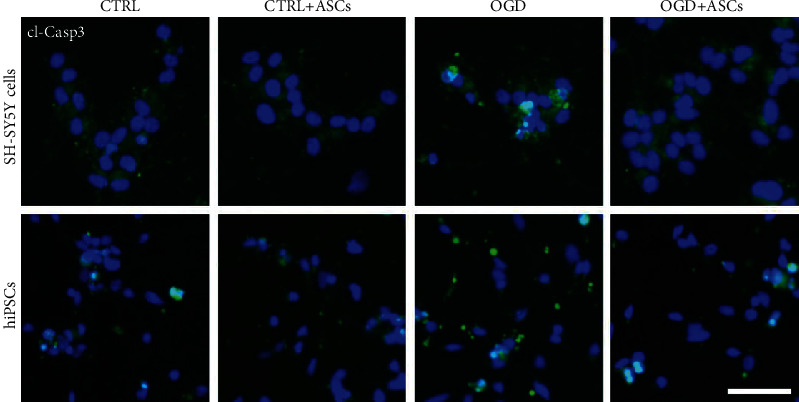
Apoptosis of SH-SY5Y- and hiPSC-neurons after OGD and ASC coculture, as determined by cleaved caspase-3 (cl-Casp3) staining. Representative images of cl-Casp3 staining after 72 h of reperfusion in SH-SY5Y-neurons (*n* = 1/group/experiment, representative image of two to four separate experiments; two to four images were taken of each well) and hiPSC-neurons (*n* = 1/group/experiment, representative images of two separate experiments; 4 images were taken of each well). Blue = nuclei; green = cl-Casp3. Scale bar, 50 *μ*m.

Then, the viability and cytotoxicity were studied with live/dead staining, and the percentages of areas with live cells and with dead cells were quantified. Overall, live/dead staining showed that SH-SY5Y-neurons were viable after OGD treatment (Figure 3(a)). However, there was a significant decrease in the percentage of live cells in the OGD group compared to the control group (*p* = 0.002) (Figure 3(b)). Similarly, after OGD (*p* = 0.001) and 24 h (*p* < 0.001) and 72 h (*p* = 0.012) after reperfusion, an increase in the number of dead cells compared to that in the respective control group was observed (Figure 3(c)). ASC treatment significantly increased the percentage of live SH-SY5Y-neurons after 72 h of culturing (*p* = 0.015) in control conditions (Figures 3(a) and 3(b)) but not after OGD at any of the timepoints. Instead, upon coculture with ASCs, a decrease in the number of dead cells was observed in both control and OGD conditions at 24 (CTRL: *p* < 0.001; OGD: *p* = 0.002) and 72 h (CTRL: *p* < 0.001; OGD: *p* = 0.008) after reperfusion (Figures 3(a) and 3(c)). CyQuant analysis showed that fewer SH-SY5Y-neurons were detected at all timepoints in OGD conditions than in control conditions (0 h: *p* = 0.036; 24 h: *p* < 0.001; 72 h: *p* < 0.001). Coculture with ASCs significantly increased the number of SH-SY5Y-neurons after 24 h of reperfusion (*p* < 0.001) in OGD conditions, while ASCs did not have an effect on the number of cells in control conditions (Figure 3(d)). Next, apoptosis was studied by cl-Casp3 staining. More cl-Casp3-positive SH-SY5Y-neurons were detected in OGD conditions than in control conditions, while fewer were detected after ASC coculture, especially after 72 h of reperfusion (Figure 4).

Similarly, as for SH-SY5Y-neurons, the viability, cytotoxicity, and apoptosis of hiPSC-neurons were studied after OGD and coculture with ASCs. Quantification of the area (Figures 3(e) and 3(f)) showed that the percentage of live cells was significantly decreased in the 24 h reperfusion group compared to the control group (*p* = 0.015), while no differences were found in the percentage of dead cells between OGD and control conditions (Figures 3(e) and 3(g)). ASC coculture significantly increased the percentage of live cells at the 72 h timepoint in OGD (*p* = 0.017) and control (*p* = 0.002) conditions and decreased the percentage of dead cells at the 72 h timepoint in control conditions (*p* < 0.001). CyQuant analysis of hiPSC-neurons illustrated that the number of these cells was decreased immediately after OGD (*p* = 0.016) and 24 h after reperfusion (*p* = 0.003) compared to after culture in control conditions, but no differences were noticed between the OGD group and the control group after 72 h of reperfusion (*p* < 0.05). Compared to control culture, coculture with ASCs increased the number of cells 72 h after reperfusion (*p* = 0.008; Figure 3(h)). However, under OGD conditions, ASC coculture decreased the number of hiPSC-neurons 24 h after reperfusion (*p* = 0.019; Figure 3(h)). In hiPSC-neurons, a higher number of cl-Casp3-positive cells were detected after OGD than after ASC treatment (Figure 4).

### 3.2. ASC Coculture Enhanced the Proliferation of SH-SY5Y- and hiPSC-Neurons in Both OGD and Control Conditions

**Figure 5:**
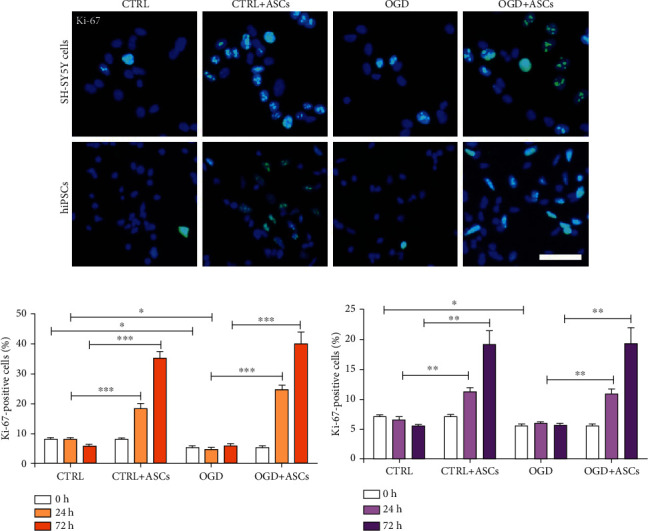
Proliferation of SH-SY5Y- and hiPSC-neurons after OGD and ASC coculture. (a) Representative images of Ki-67 staining after OGD and 72 h after reperfusion in SH-SY5Y- and hiPSC-neurons. Blue = nuclei; green = Ki-67. Scale bar, 50 *μ*m. (b) Quantification (%) of Ki-67-positive SH-SY5Y- and (c) hiPSC-neurons. Two to four representative images (two to four independent experiments) of SH-SY5Y-neurons and 4 representative images (two independent experiments) of hiPSC-neurons were analyzed. All statistical analyses were performed using an independent *t*-test, statistical significance: ^∗∗∗^*p* < 0.001, ^∗∗^*p* < 0.01, and ^∗^*p* < 0.05. All quantitative data are presented as the mean ± SEM.

To investigate the effect of OGD and ASC coculture on proliferation, cells were stained with the cell cycle marker Ki-67 and quantified. The number of Ki-67-positive SH-SY5Y-neurons was decreased after OGD (*p* = 0.011) and 24 h after reperfusion (*p* = 0.017) compared to that after culture in control conditions. Coculture with ASC significantly increased the percentage of Ki-67-positive cells in both control and OGD conditions after 24 (CTRL: *p* < 0.001; OGD: *p* < 0.001) and 72 h (CTRL: *p* < 0.001; OGD: *p* < 0.001) of reperfusion (Figures 5(a) and 5(b)). A decreased number of Ki-67-positive hiPSC-neurons were detected after OGD (*p* = 0.013) compared to that after control culture, whereas after 24 and 72 h of reperfusion, the number of Ki-67-positive cells was the same as that in the control group. Coculture with ASC increased the percentage of Ki-67-positive cells in both the control and OGD groups after 24 (CTRL: *p* = 0.002; OGD: *p* = 0.001) and 72 h (CTRL: *p* = 0.012; OGD: *p* = 0.014) of reperfusion (Figures 5(a) and 5(c)).

### 3.3. Axonal Damage to SH-SY5Y- and hiPSC-Neurons Was Increased in OGD Conditions

**Figure 6:**
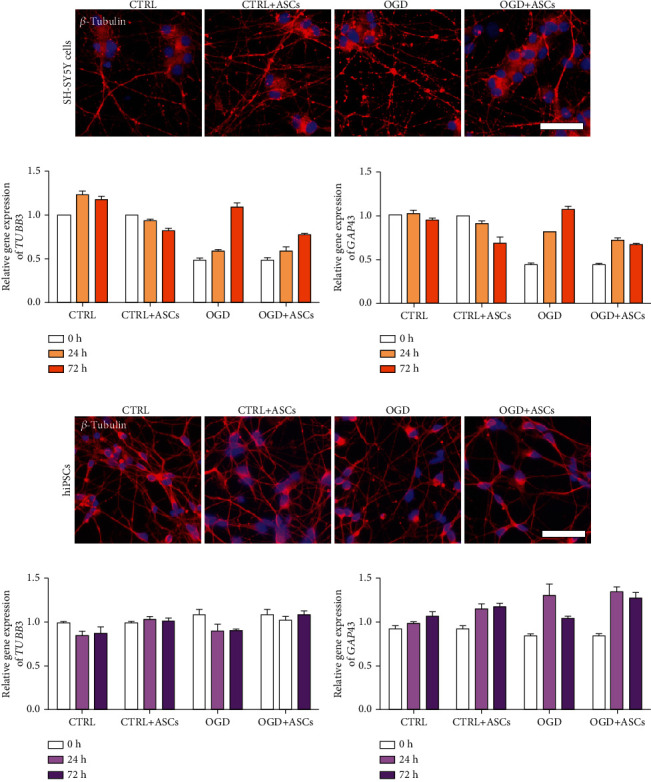
Axonal damage to SH-SY5Y- and hiPSC-neurons. (a, d) Representative images of *β*-tubulin_III_ staining after 72 h of reperfusion in (a) SH-SY5Y- and (d) hiPSC-neurons (blue = nuclei, red = *β*-tubulin_III_), scale bar, 50 *μ*m (representative images of two to four separate experiments for SH-SY5Y-neurons and two separate experiments for hiPSC-neurons). (b, c) Relative expression of *TUBB3* and *GAP43* in SH-SY5Y-neurons (*n* = 1 from one experiment). (e, f) Relative expression of *TUBB3* and *GAP43* in hiPSC-neurons after OGD and 24 h or 72 h of reperfusion (*n* = 2 from two experiments).

To examine whether OGD disrupts the integration of microtubules in axons, the expression of *β*-tubulin_III_ was studied at the gene and protein levels. Furthermore, gene expression of the neuronal growth cone marker *GAP43* was used to detect regenerative responses after OGD. In SH-SY5Y-neurons, the relative expression of *TUBB3* was decreased after OGD and 24 h of reperfusion compared to after control culture and then returned to the control level. Coculture with ASC did not influence the expression of *TUBB3* (Figure 6(b)). *β*-Tubulin_III_ was expressed along the cytoskeleton of the neurons under control conditions. After OGD, *β*-tubulin_III_ staining became fragmented, showing disintegration in axons. Upon coculture with ASCs, less axonal disintegration of *β*-tubulin_III_ SH-SY5Y-neurons was observed in OGD conditions compared to control conditions (Figure 6(a)). The relative expression of *GAP43* was decreased after OGD and 24 h of reperfusion compared to after control culture, whereas no effect of ASCs on its expression levels was detected (Figure 6(c)). In hiPSC-neurons, the relative gene expression of *TUBB3* was not different between the OGD group and the control group (Figure 6(e)), while *β*-tubulin_III_ was expressed at a lower level around the cell body and along the axons (Figure 6(d)) in OGD conditions compared to control conditions. Axons were denser in ASC and hiPSC-neuron cocultures than in OGD conditions. The relative gene expression of *GAP43* was similar between the control and OGD groups, whereas its expression increased upon ASC coculture in OGD conditions (Figure 6(f)) compared to that in OGD conditions alone.

## 4. Discussion

Stroke has been modeled *in vitro* by removing oxygen and glucose from the cells or by chemical or enzymatic inhibition of cellular metabolism [7]. Most frequently, ischemia-like conditions are produced with OGD conditions, which involve replacing O_2_/CO_2_ equilibrated medium with N_2_/CO_2_ equilibrated glucose-free medium and maintaining cells in a hypoxic atmosphere [7, 40]. Since neurons have high glucose and oxygen demands [41], the removal of glucose and oxygen leads to impairment in maintaining normal ionic gradients followed by excitotoxicity, oxidative stress, and eventually apoptosis, autophagocytosis, and necrotic cell death [42, 43]. Commonly, OGD is followed by a reperfusion period, in which the restoration of glucose and oxygen to the cells causes the production of reactive oxygen species, which further induce cellular damage [44] and neuronal degeneration [45]. There is no standardized OGD protocol to mimic stroke *in vitro*, and in previous studies, the duration of OGD has ranged from 1 to 24 h [46] with or without a subsequent reperfusion period. The O_2_ concentration in hypoxic environments varies from 0% [47–53] to 8% [54]. In our *in vitro* stroke model, 24 h of OGD with 1% O_2_ and 24 or 72 h of reperfusion period was used.

Cellular platforms that have been used to model stroke *in vitro* include brain slices, organotypic cell cultures, primary neuronal cells, immortalized cell lines, and stem cells of human and rodent origin [7]. Human brain slices and primary cells are highly physiologically relevant; however, they are extremely limited in availability [7]; therefore, most OGD studies are performed with primary rodent neuronal cells or human neuroblastoma cell lines such as the SH-SY5Y cell line [9]. Prior studies in SH-SY5Y cells have used either undifferentiated cells or cells differentiated into neuronal cells to more precisely mimic a mature neuronal phenotype [9, 11]. However, high passage numbers and oncogenes limit the physiological relevance of neuroblastoma cell lines [7]. Human stem cells have a high potential to be utilized for *in vitro* stroke models because they are unlimited and have the potential for efficient neuronal differentiation [55]. hiPSC-derived neuronal networks have shown similar patterns of functionality as primary rat cells [32]. Currently, no studies have used hiPSC-derived neurons to model stroke *in vitro*. Here, two different cell types of human origin, SH-SY5Y cells and hiPSCs, were differentiated into neurons and used to model stroke *in vitro*. SH-SY5Y cells were differentiated into neurons using gradual serum starvation and the addition of extracellular matrix proteins and neurotrophic factors to yield more homogenous and mature neuronal cultures [9], while hiPSCs were differentiated into cortical cultures with small molecules to yield mature, functionally active neurons [32].

In our study, cytotoxicity and apoptosis of SH-SY5Y-neurons were increased, and viability SH-SY5Y-neurons was decreased after OGD followed by 24 or 72 h of reperfusion. This is in line with previous studies in nondifferentiated SH-SY5Y cells showing decreased cell viability and activation of apoptosis after OGD [53, 56]. Gao et al. showed that cell viability decreases, and the apoptosis rate increases after 16 h of OGD and 9 h of reperfusion. Lee et al. showed that cell viability decreases after 20 h of OGD and 24 h of reperfusion in SH-SY5Y cells. Similar to SH-SY5Y-neurons, hiPSC-neurons showed increased cytotoxicity and apoptosis after OGD and 24 h of reperfusion, although the magnitude of damage was not as robust as in SH-SY5Y-neurons. In hiPSC-neurons, the cytotoxic effect of OGD was detected by determining the number of cells with CyQuant analysis but not with live/dead staining; however, OGD-induced damage was detected by both methods in SH-SY5Y-neurons. Altogether, our results show that both cell models respond to the same OGD parameters rather similarly, suggesting that the OGD paradigm can be standardized between different cell types used to model stroke *in vitro*.

Ischemic stroke causes rapid and significant loss of axons in the brain [57]. Thus, we explored *in vitro* axonal damage after OGD in both cell models. SH-SY5Y-neurons showed more disintegrated axons than hiPSC-neurons, although the morphology of hiPSC-neurons was changed after OGD compared to after control culture. Consistent with our studies, others have reported severe axonal damage in primary rodent neurons after OGD [50, 52]. Liu et al. showed degradation and disappearance of axons, a decrease in the length of the axons, and a change in the morphology of primary rat neurons after 90 min of OGD [52]. A similar effect was also shown in primary hamster neuronal hippocampal cells after 2 h of OGD and 48 h of reperfusion; neurons were severely injured, and axonal processes disappeared [50]. It seems that in primary neurons compared to SH-SY5Y- or hiPSC-neurons, OGD-induced cell damage is more rapid and more destructive to axons. We also studied the effects of OGD-induced axonal damage on the gene expression of the microtubule protein *TUBB3* and the neuronal growth cone marker *GAP43*, which is considered an essential player in regenerative responses in the CNS [58]. In our study, SH-SY5Y-neurons showed decreased expression of *GAP43* and *TUBB3* after OGD and 24 h of reperfusion. Similar effects were also reported for GAP43 protein expression in rat cortical neurons after OGD [52]. However, in hiPSC-neurons, a similar effect was not observed, as OGD did not influence the expression of *GAP43* or *TUBB3*. Overall, SH-SY5Y-neurons were more prone to OGD-induced damage, while injury was less severe in hiPSC-neurons. The limited responsiveness of hiPSC-neurons to OGD might be due to the plasticity of hiPSCs. Most hiPSC-derived cells mimic the embryonic or fetal stage of development and therefore have a more robust ability to overcome damage [14]. Moreover, culture conditions, such as cell density and the composition of the medium used, may also influence the severity of damage.

After setting up the *in vitro* OGD-induced models in SH-SY5Y- and hiPSC-neurons, they were used to study the potential neuroprotective effect of ASCs. Studies in animal models of stroke have shown that MSC treatment has beneficial effects on stroke recovery; however, contradictory results have also been reported [16]. The majority of *in vitro* and *in vivo* studies have been performed on rodent and human BM-MSCs, while human-derived ASCs are less studied [15]. Here, ASCs were cocultured on inserts with SH-SY5Y- and hiPSC-neurons after OGD for 24 or 72 h. The results showed that ASC coculture increased cell viability and decreased cytotoxicity and cell death in both cell models. Similar findings have been reported for BM-MSCs [47, 48, 51, 54] and ASCs [59, 60] in various cell types, suggesting the overall beneficial effects of ASCs after OGD.

MSCs have been shown to provide neuroprotection by inhibiting apoptosis after OGD [47, 48, 50, 51, 54]. This antiapoptotic effect has been observed in neurons treated either with human or mouse BM-MSCs or their conditioned medium prior to OGD [50, 51] or during reperfusion [47, 48]. In our study, apoptosis was evaluated with cl-Casp3 staining, which showed that ASC coculture decreased the number of cl-Casp3-positive SH-SY5Y- and hiPSC-neurons after OGD. Similar findings have been reported in rat primary neurons after treatment with ASC conditioned medium or coculture with BM-MSCs for 24-48 h after OGD [48, 60].

MSCs secrete multiple soluble factors, such as cytokines, chemokines, and growth factors [19], which have an impact on the proliferation of neurons. Interestingly, in our study, ASC coculture increased the proliferation of SH-SY5Y- and hiPSC-neurons 24 and 72 h after OGD. Similar to our findings, increased proliferation of BM-MSC-treated human neuroblastoma M17 cells was observed after 24 and 48 h of reperfusion but not after 72 h of reperfusion [47]. Additionally, BM-MSCs have been shown to promote the proliferation of endogenous neural stem cells in a rat stroke model [61]. Here, increased proliferation after ASC coculture may also have been related to the presence of mitogenic factors in the ASC medium. Thus, the proliferative response of SH-SY5Y- and hiPSC-neurons seen here might have been due to the combined effect of mitogenic factors and secreted factors from ASCs.

Treatment with human BM-MSCs has been shown to promote axonal outgrowth, increase the length of axons [52], and diminish axonal disintegration [50] in primary rodent neurons after OGD-induced injury. In the present study, axons seemed less disintegrated after ASC coculture than after OGD, as demonstrated by *β*-tubulin_III_ staining. In SH-SY5Y-neurons, the gene expression of *TUBB3* and *GAP43* was not changed after ASC coculture, while *GAP43* levels were increased 72 h after reperfusion in hiPSC-neurons, indicating ongoing regenerative processes. Similarly, increased GAP43 protein expression was observed by Liu et al. in rat cortical neurons after 48 h of exposure to both human BM-MSCs and their conditioned medium during reperfusion [52].

Overall, SH-SY5Y-neurons responded more strongly to OGD-induced injury and were more affected by subsequent ASC coculture than hiPSC-neurons. Surprisingly, ASC coculture appeared to be somewhat harmful to OGD-challenged hiPSC-neurons when cell numbers were compared using CyQuant analysis. In contrast, under control conditions, ASCs increased the number and viability of hiPSC-neurons. The harmful effect of ASCs after OGD injury might have been due to factors secreted by ASCs or because hiPSC-neurons were unable to adapt to the ASC microenvironment after OGD insult. This further suggests that OGD-treated hiPSC-neurons are more vulnerable to microenvironmental changes than control hiPSC-neurons. ASCs were grown on inserts that allowed trophic factors to diffuse between the cultures. Sheibe et al. reported that high concentrations (over 10%) of conditioned medium from mouse and human BM-MSCs and coculture with a high number of BM-MSCs have toxic effects on mouse primary neuronal cells [51]. In the present study, 10,000 ASCs were used for both neuronal cell types, which was twice the cell number that Sheibe et al. reported to be toxic. ASCs might also use the nutrients in the medium that are required by the neurons or secrete trophic factors that are harmful to OGD-treated hiPSC-neurons.

## 5. Conclusions

In conclusion, our data suggest that both cell types, SH-SY5Y- and hiPSC-neurons, are suitable for modeling stroke *in vitro*. Both cell types responded to OGD treatment; however, OGD had a stronger effect on SH-SY5Y-neurons than on hiPSC-neurons with immature phenotype. Our data also suggest that ASCs have neuroprotective effects after OGD injury in SH-SY5Y-neurons and hiPSC-neurons *in vitro*.

## Data Availability

The datasets generated during and/or analyzed during the current study are available from the corresponding author on reasonable request.

## References

[1] Rajsic S., Gothe H., Borba H. H. (2019). Economic burden of stroke: a systematic review on post-stroke care. *The European Journal of Health Economics*.

[2] George P. M., Steinberg G. K. (2015). Novel stroke therapeutics: unraveling stroke pathophysiology and its impact on clinical treatments. *Neuron*.

[3] Donnan G. A., Davis S. M., Parsons M. W., Ma H., Dewey H. M., Howells D. W. (2011). How to make better use of thrombolytic therapy in acute ischemic stroke. *Nature Reviews Neurology*.

[4] Bhaskar S., Stanwell P., Cordato D., Attia J., Levi C. (2018). Reperfusion therapy in acute ischemic stroke: dawn of a new era?. *BMC Neurology*.

[5] Kleinschnitz C., Fluri F., Schuhmann M. (2015). Animal models of ischemic stroke and their application in clinical research. *Drug Design, Development and Therapy*.

[6] Borlongan C. V. (2019). Concise review: stem cell therapy for stroke patients: are we there yet?. *Stem Cells Translational Medicine*.

[7] Holloway P. M., Gavins F. N. E. (2016). Modeling ischemic stroke in vitro: status quo and future perspectives. *Stroke*.

[8] Liu Y., Eaton E. D., Wills T. E., McCann S. K., Antonic A., Howells D. W. (2018). Human ischaemic cascade studies using SH-SY5Y cells: a systematic review and meta-analysis. *Translational Stroke Research*.

[9] Shipley M. M., Mangold C. A., Szpara M. L. (2016). Differentiation of the SH-SY5Y human neuroblastoma cell line. *Journal of Visualized Experiments*.

[10] Agholme L., Lindström T., Kågedal K., Marcusson J., Hallbeck M. (2010). An in vitro model for neuroscience: differentiation of SH-SY5Y cells into cells with morphological and biochemical characteristics of mature neurons. *Journal of Alzheimer's Disease*.

[11] Kovalevich J., Langford D. (2013). Considerations for the use of SH-SY5Y Neuroblastoma Cells in Neurobiology. *Methods in Molecular Biology*.

[12] Xicoy H., Wieringa B., Martens G. J. M. (2017). The SH-SY5Y cell line in Parkinson's disease research: a systematic review. *Molecular Neurodegeneration*.

[13] Hunsberger J. G., Efthymiou A. G., Malik N. (2015). Induced pluripotent stem cell models to enable in vitro models for screening in the central nervous system. *Stem Cells and Development*.

[14] Doss M. X., Sachinidis A. (2019). Current challenges of iPSC-based disease modeling and therapeutic implications. *Cells*.

[15] Fernández-Susavila H., Bugallo-Casal A., Castillo J., Campos F. (2019). Adult stem cells and induced pluripotent stem cells for stroke treatment. *Frontiers in Neurology*.

[16] Laso-García F., Diekhorst L., Gómez-de Frutos M. C. (2019). Cell-based therapies for stroke: promising solution or dead end? Mesenchymal stem cells and comorbidities in preclinical stroke research. *Frontiers in Neurology*.

[17] Wang F., Tang H., Zhu J., Zhang J. H. (2018). Transplanting mesenchymal stem cells for treatment of ischemic stroke. *Cell Transplant*.

[18] Cui L., Golubczyk D., Tolppanen A., Boltze J., Jolkkonen J. (2019). Cell therapy for ischemic stroke: are differences in preclinical and clinical study design responsible for the translational loss of efficacy?. *Annals of Neurology*.

[19] Fu Y., Karbaat L., Wu L., Leijten J., Both S. K., Karperien M. (2017). Trophic effects of mesenchymal stem cells in tissue regeneration. *Tissue Engineering Part B: Reviews*.

[20] Ottoboni L., Merlini A., Martino G. (2017). Neural stem cell plasticity: advantages in therapy for the injured central nervous system. *Frontiers in Cell and Development Biology*.

[21] Berebichez-Fridman R., Montero-Olvera P. R. (2018). Sources and clinical applications of mesenchymal stem cells: state-of-the-art review. *Sultan Qaboos University Medical Journal*.

[22] Marrazzo P., Paduano F., Palmieri F., Marrelli M., Tatullo M. (2016). Highly EfficientIn VitroReparative behaviour of dental pulp stem cells cultured with standardised platelet lysate supplementation. *Stem Cells International*.

[23] Cowper M., Frazier T., Wu X. (2019). Human platelet lysate as a functional substitute for fetal bovine serum in the culture of human adipose derived stromal/stem cells. *Cells*.

[24] Gutiérrez-Fernández M., Rodríguez-Frutos B., Ramos-Cejudo J. (2013). Effects of intravenous administration of allogenic bone marrow- and adipose tissue-derived mesenchymal stem cells on functional recovery and brain repair markers in experimental ischemic stroke. *Stem Cell Research & Therapy*.

[25] on behalf of RESSTORE consortium, Gómez-de Frutos M. C., Laso-García F. (2019). Intravenous delivery of adipose tissue-derived mesenchymal stem cells improves brain repair in hyperglycemic stroke rats. *Stem Cell Research & Therapy*.

[26] Mu J., Bakreen A., Juntunen M. (2019). Combined adipose tissue-derived mesenchymal stem cell therapy and rehabilitation in experimental stroke. *Frontiers in Neurology*.

[27] Mangin G., Cogo A., Moisan A. (2019). Intravenous administration of human adipose derived-mesenchymal stem cells is not efficient in diabetic or hypertensive mice subjected to focal cerebral ischemia. *Frontiers in Neuroscience*.

[28] Diekhorst L., Gómez-de Frutos M. C., Laso-García F. (2020). Mesenchymal stem cells from adipose tissue do not improve functional recovery after ischemic stroke in hypertensive rats. *Stroke*.

[29] Kiamehr M., Klettner A., Richert E. (2019). Compromised barrier function in human induced pluripotent stem-cell-derived retinal pigment epithelial cells from type 2 diabetic patients. *International Journal of Molecular Sciences*.

[30] Ojala M., Prajapati C., Pölönen R. (2016). Mutation-specific phenotypes in hiPSC-derived cardiomyocytes carrying either myosin-binding protein C or *α*-tropomyosin mutation for hypertrophic cardiomyopathy. *Stem Cells International*.

[31] Hongisto H., Ilmarinen T., Vattulainen M., Mikhailova A., Skottman H. (2017). Xeno- and feeder-free differentiation of human pluripotent stem cells to two distinct ocular epithelial cell types using simple modifications of one method. *Stem Cell Research & Therapy*.

[32] Hyvärinen T., Hyysalo A., Kapucu F. E. (2019). Functional characterization of human pluripotent stem cell-derived cortical networks differentiated on laminin-521 substrate: comparison to rat cortical cultures. *Scientific Reports*.

[33] Lappalainen R. S., Salomäki M., Ylä-Outinen L. (2010). Similarly derived and cultured hESC lines show variation in their developmental potential towards neuronal cells in long-term culture. *Regenerative Medicine*.

[34] Carpenter A. E., Jones T. R., Lamprecht M. R. (2006). CellProfiler: image analysis software for identifying and quantifying cell phenotypes. *Genome Biology*.

[35] Jones T. R., Kang I. H., Wheeler D. B. (2008). CellProfiler Analyst: data exploration and analysis software for complex image-based screens. *BMC Bioinformatics*.

[36] Tirkkonen L., Haimi S., Huttunen S. (2013). Osteogenic medium is superior to growth factors in differentiation of human adipose stem cells towards bone-forming cells in 3D culture. *European Cells & Materials*.

[37] Vanhatupa S., Ojansivu M., Autio R., Juntunen M., Miettinen S. (2015). Bone morphogenetic protein-2 induces donor-dependent osteogenic and adipogenic differentiation in human adipose stem cells. *Stem Cells Translational Medicine*.

[38] Pfaffl M. W. (2001). A new mathematical model for relative quantification in real-time RT-PCR. *Nucleic Acids Research*.

[39] Hyvärinen T., Hagman S., Ristola M. (2019). Co-stimulation with IL-1*β* and TNF-*α* induces an inflammatory reactive astrocyte phenotype with neurosupportive characteristics in a human pluripotent stem cell model system. *Scientific Reports*.

[40] Woodruff T. M., Thundyil J., Tang S., Sobey C. G., Taylor S. M., Arumugam T. V. (2011). Pathophysiology, treatment, and animal and cellular models of human ischemic stroke. *Molecular Neurodegeneration*.

[41] Mergenthaler P., Lindauer U., Dienel G. A., Meisel A. (2013). Sugar for the brain: the role of glucose in physiological and pathological brain function. *Trends in Neurosciences*.

[42] Lipton P. (1999). Ischemic cell death in brain neurons. *Physiological Reviews*.

[43] Mehta S. L., Manhas N., Raghubir R. (2007). Molecular targets in cerebral ischemia for developing novel therapeutics. *Brain Research Reviews*.

[44] Cowled P., Fitridge R., Fitridge R., Thompson M. (2011). Pathophysiology of reperfusion injury. *Mechanisms of Vascular Disease: A Reference Book for Vascular Specialists*.

[45] Goldberg M. P., Choi D. W. (1993). Combined oxygen and glucose deprivation in cortical cell culture: calcium-dependent and calcium-independent mechanisms of neuronal injury. *The Journal of Neuroscience*.

[46] Sommer C. J. (2017). Ischemic stroke: experimental models and reality. *Acta Neuropathologica*.

[47] Huang P., Gebhart N., Richelson E., Brott T. G., Meschia J. F., Zubair A. C. (2014). Mechanism of mesenchymal stem cell-induced neuron recovery and anti-inflammation. *Cytotherapy*.

[48] Kong D., Zhu J., Liu Q. (2017). Mesenchymal stem cells protect neurons against hypoxic-ischemic injury via inhibiting parthanatos, necroptosis, and apoptosis, but not autophagy. *Cellular and Molecular Neurobiology*.

[49] Gu Y., He M., Zhou X. (2016). Endogenous IL-6 of mesenchymal stem cell improves behavioral outcome of hypoxic-ischemic brain damage neonatal rats by supressing apoptosis in astrocyte. *Scientific Reports*.

[50] Piscioneri A., Morelli S., Mele M. (2015). Neuroprotective effect of human mesenchymal stem cells in a compartmentalized neuronal membrane system. *Acta Biomaterialia*.

[51] Scheibe F., Klein O., Klose J., Priller J. (2012). Mesenchymal stromal cells rescue cortical neurons from apoptotic cell death in an in vitro model of cerebral ischemia. *Cellular and Molecular Neurobiology*.

[52] Liu Y., Zhang Y., Lin L. (2013). Effects of bone marrow-derived mesenchymal stem cells on the axonal outgrowth through activation of PI3K/AKT signaling in primary cortical neurons followed oxygen-glucose deprivation injury. *PLoS One*.

[53] Lee H. J., Lyu D. H., Koo U. (2011). Inhibitory effect of 2-arylbenzofurans from the Mori Cortex Radicis (Moraceae) on oxygen glucose deprivation (OGD)-induced cell death of SH-SY5Y cells. *Archives of Pharmacal Research*.

[54] Zhang Y., Yu S., Tuazon J. P. (2019). Neuroprotective effects of human bone marrow mesenchymal stem cells against cerebral ischemia are mediated in part by an anti-apoptotic mechanism. *Neural Regeneration Research*.

[55] Selvaraj V., Jiang P., Chechneva O., Lo U.-G., Deng W. (2012). Differentiating human stem cells into neurons and glial cells for neural repair. *Frontiers in Bioscience*.

[56] Gao G., Li Y., Zhai H. (2017). Humanin analogue, S14G-humanin, has neuroprotective effects against oxygen glucose deprivation/reoxygenation by reactivating Jak2/Stat3 signaling through the PI3K/AKT pathway. *Experimental and Therapeutic Medicine*.

[57] Hinman J. D. (2014). The back and forth of axonal injury and repair after stroke. *Current Opinion in Neurology*.

[58] Holahan M. R. (2017). A shift from a pivotal to supporting role for the growth-associated protein (GAP-43) in the coordination of axonal structural and functional plasticity. *Frontiers in Cellular Neuroscience*.

[59] Jeon D., Chu K., Lee S. (2013). Neuroprotective effect of a cell-free extract derived from human adipose stem cells in experimental stroke models. *Neurobiology of Disease*.

[60] Du S., Mao G., Zhu T., Luan Z., Du Y., Gu H. (2015). TIMP1 in conditioned media of human adipose stromal cells protects neurons against oxygen-glucose deprivation injury. *Neuroscience Letters*.

[61] Yoo S., Kim S., Lee S. (2008). Mesenchymal stem cells promote proliferation of endogenous neural stem cells and survival of newborn cells in a rat stroke model. *Experimental and Molecular Medicine*.

